# Clinical illness and outcomes in Nigerian children with late-appearing anaemia after artemisinin-based combination treatments of uncomplicated falciparum malaria

**DOI:** 10.1186/s12879-016-1565-4

**Published:** 2016-06-01

**Authors:** Akintunde Sowunmi, Kazeem Akano, Adejumoke I. Ayede, Godwin Ntadom, Temitope Aderoyeje, Elsie O. Adewoye, Bayo Fatunmbi

**Affiliations:** Department of Pharmacology and Therapeutics, University of Ibadan, Ibadan, Nigeria; Institute for Medical Research and Training, University of Ibadan, Ibadan, Nigeria; Department of Paediatrics, University of Ibadan, Ibadan, Nigeria; National Malaria Elimination Programme, Federal Ministry of Health, Abuja, Nigeria; Department of Clinical Pharmacology, University College Hospital, Ibadan, Nigeria; Department of Physiology, University of Ibadan, Ibadan, Nigeria; World Health Organization, Regional Office for the Western Pacific, Phnom Penh, Cambodia

**Keywords:** Late-appearing anaemia, Artemisinin-based combination treatments, Children, Nigeria

## Abstract

**Background:**

Late-appearing anaemia (LAA) following treatment with artemisinins for severe malaria has been reported and well described, but there are limited clinical and parasitological data on LAA in African children with uncomplicated falciparum malaria following oral artemisinin-based combination therapies (ACTs).

**Methods:**

This was an open label study with the main objectives of evaluating the clinical features, the risk factors for, the temporal changes in haematocrit and the outcomes of a LAA in malarious children treated with artesunate-amodiaquine (AA), artemether-lumefantrine (AL) or dihydroartemisinin-piperaquine (DHP). The diagnosis of LAA was made using the criteria: clearance of parasitaemia, fever and other symptoms within 1 week of commencing treatment; adequate clinical and parasitological response at 4–6 weeks after treatment began; haematocrit ≥30 % 1 and/or 2 weeks after treatment began; and haematocrit <30 %, parasite negativity by microscopy and polymerase chain reaction and absence of concomitant illness 3–6 weeks after treatment began.

**Results:**

LAA occurred in 84 of 609 children, was mild, moderate or severe in 77, 6 or 1 child, respectively and was relatively asymptomatic. Mean time elapsing from commencement of treatment to LAA was 27.1 days (95 % CI 25.3–28.9). In a multivariate analysis, an age <3 years (adjusted odd ratio [AOR] = 2.6, 95 % CI 1.3–5.2, *P* = 0.005), fever 1 day after treatment began (AOR = 3.8, 95 % CI 1.8–8.2, *P* < 0.0001), haematocrit <25 % at presentation (AOR = 2.2, 95 % CI 1.3–3.7, *P* = 0.003), haematocrit <30 % 1 day after treatment began (AOR = 2.1, 95 % CI 1.0–4.3, *P* = 0.04), parasite reduction ratio >10^4^ 2 days after treatment began (AOR = 2.1, 95 % CI 1.1–3.9, *P* = 0.03) and spleen enlargement at presentation (AOR = 2.0, 95 % CI 1.1–3.9, *P* < 0.0001) were independent predictors of LAA. During 6 weeks of follow-up, uneventful recovery from anaemia occurred in 56 children [mean recovery time of 11.8 days (95 % CI 10.3–13.3)]. The only independent predictor of failure of recovery was LAA occurring 4 weeks after starting treatment (AOR = 7.5, 95 % CI 2.5–22.9, *P* < 0.0001).

**Conclusion:**

A relatively asymptomatic LAA with uneventful recovery can occur in young malarious children following ACTs. Its occurrence may have implications for case and community management of anaemia and for anaemia control efforts in sub–Saharan Africa where ACTs have become first-line antimalarials.

**Trials registration:**

Pan African Clinical Trial Registry PACTR201508001188143, 3 July 2015; PACTR201510001189370, 3 July 2015; PACTR201508001191898, 7 July 2015 and PACTR201508001193368, 8 July 2015 http://www.pactr.org.

## Background

Haemolytic anaemia is an inevitable consequence of *Plasmodium falciparum* infections. It is due to destruction of parasitized and non-parasitized red blood cells and bone marrow dyserythropoeisis of varying intensity and duration [1–4]. Following treatment with artemisinin-like drugs, dead parasites are removed from infected red blood cells during passage through the spleen by ‘pitting’ [5, 6]. These once-infected red blood cells have relatively short life-span and are destroyed 7–21 days later [6, 7]. In immunologically naïve patients with severe malaria, intravenous artesunate treatment may cause a severe form of delayed haemolytic anaemia – the postartesunate delayed haemolysis (PADH) syndrome [7–9]. The case definition for PADH syndrome is: 10 % fall in pre-treatment haemoglobin associated with haptoglobin <0.1 g/L and either an increase in lactate dehydrogenase (LDH) to >390 IL/L or a 10 % rise >7 days after start of treatment [7, 10]. This syndrome has been reported following intravenous artesunate treatment of severe malaria in 1–7 % of African children [11, 12].

The features of non-severe and of severe malaria differ considerably in children [13, 14]. Certain features of severe malaria in children resident in endemic areas may be considerably modified by immunity. For example, hyperparasitaemia, considered a feature of severe malaria [15], is not a reliable indicator of severity or poor prognosis in children resident in endemic areas [14, 16]. In addition, hyperparasitaemia not accompanied by other features of severe malaria, may be treated with oral artemisinin-based combinations [17, 18]. Although PADH syndrome can occur in uncomplicated falciparum malaria after oral artemisinin-based combination treatments [19], there is little characterisation of the clinical and parasitological features of the recently described late-appearing form of post artemisinin-based combination treatments-related anaemia in African children with uncomplicated falciparum malaria [20]. Such information may not only assist with case and community management of malaria-related anaemia but also in defining the epidemiology and the risk factors associated with the late-appearing form of post artemisinin-based combination treatments-related anaemia.

In the present study, using the criteria: clearance of parasitaemia, fever and other symptoms within 1 week of commencing treatment; adequate clinical and parasitological response at 4–6 weeks after treatment began; haematocrit ≥30 % 1 and/or 2 weeks after treatment began; and haematocrit <30 %, parasite negativity by microscopy and polymerase chain reaction (PCR), and absence of concomitant illness 3–6 weeks after treatment began, we described in children with symptomatic uncomplicated falciparum malaria, the clinical illness and outcomes of a relatively asymptomatic late-appearing anaemia (LAA) following artesunate-amodiaquine, artemether-lumefantrine or dihydroartemisinin-piperaquine treatments in a subset of 609 malarious children in an endemic area. The main aims of the study were, using these criteria, to: (i) determine the frequency of LAA, (ii) describe the clinical illness and the outcomes of LAA, and (iii) evaluate the factors contributing to LAA and its outcomes in malarious children in an endemic area, following artemisinin-based combination treatments of uncomplicated infections.

## Methods

### Study location

The study was an open label randomised study in children with uncomplicated falciparum malaria who were treated with artesunate-amodiaquine (AA), artemether-lumefantrine (AL) and dihydroartemisinin-piperaquine (DHP) between January 2008 and December 2014 in Ibadan, south western Nigeria. In this area, malaria is hyperendemic and transmission occurs all year round; however, it is more intense during the raining season from April to October. Children are more affected than adults. Randomisation to AA and AL was from 2008–2013 (initial study) at a ratio of 3:1, respectively. The later study was from January to December 2014; randomization was to AA, AL and DHP at a ratio of 1:1:2, respectively. The study protocol was approved by The Ethics Committee of The Ministry of Health, Ibadan and by National Health Research Ethics Committee, Abuja, Nigeria [Pan African Clinical Trial Registry PACTR201508001188143; PACTR201510001189370; PACTR201508001191898; PACTR201508001193368].

### Patients

#### Inclusion and exclusion criteria

Patients were enrolled in the study if they met the following criteria: age 6 months–15 years, symptoms compatible with acute uncomplicated malaria with *Plasmodium falciparum* mono-infection ≥1000 μL^−1^ of blood, no history of antimalarial drug ingestion in the 2 weeks prior to enrolment, absence of severe malaria [13–15], written informed consent given by parents or guardians and ability to comply with a 42-day follow-up period. Patients with uncomplicated hyperparasitaemia (parasitaemia >250,000 μL^−1^) were not excluded from the study [14]. Patient with severe malnutrition (i. e. weight for age <60 % and bilateral oedema) and those with sickle cell anaemia were excluded from the study. Patient selection and enrolment were done by a physician who did not participate in patient evaluation once treatment began.

#### Drug treatment

Patients were randomised to drug treatment using a table of random numbers. Randomised treatment for each patient was kept in a sealed, opaque envelope. Each envelop was opened at the time of treatment by the attending nurse or physician. Enrolled patients were randomised to, and received artemether-lumefantrine, artesunate-amodiaquine (co-formulated) or dihydroartemisinin-piperaquine (co-formulated). All drugs were given orally according to body weight for 3 days (Table 1). Each tablet of artemether-lumefantrine (Coartem®, Novatis, Basel, Switzerland) contains 20 mg of artemether and 120 mg of lumefantrine. Each tablet of dihydroartemisinin-piperaquine (Duo-cotecxin®, Zhejiang Holley Nanhu, China) contains 40 mg of dihydroartemisinin and 320 mg of piperaquine. The formulations artesunate-amodiaquine (Coarsucam®, Sanofi Aventis, France) are 25 mg/67.5 mg, 50 mg/135 mg, 100 mg/270 mg of fixed dose combination.

**Table 1:** Treatment regimens

Artemether-lumefantrine	
Patients weighing:	
5–14 kg received 1 tablet,	
15–24 kg received 2 tablets,	
25–34 kg received 3 tablets,	
> 34 kg received 4 tablets at presentation (0 h), 8 h later and at 24, 36, 48 and 60 h after the first dose.	
Artesunate-amodiaquine	
Patients weighing:	
≥ 4.5 – < 9 kg received 1 tablet of 25 mg/67.5 mg formulation	
≥ 9 – < 18 kg received 1 tablet of 50 mg/135 mg formulation	
≥ 18 – < 24 kg received 1 tablet of 100 mg/270 mg formulation	
24–36 kg received 1.5 tablet of 100 mg/270 mg formulation	
> 36 kg received 2 tablets of 100/270 mg formulation daily for 3 days.	
Dihydroartemisinin-piperaquine	
Patients weighing:	
≥4.5 – <10 kg received ¾ of 1 tablet	
10 – <16 kg, received 1.5 tablet	
16 – <24 kg, received 2 tablets	
24 – <34 or received 2.5 tablets	
34 – <50 kg received 3 tables daily for 3 days.	

All doses of artesunate-amodiaquine and dihydroartemisinin-piperaquine were given under direct observed therapy as were the doses of artemether-lumefantrine given at 0, 8, 24 and 48 h. Doses of artemether-lumefantrine at 36 and 60 h were given by parents/guardians of the children at home and enquiries were made by telephone calls at the expected times of administration to confirm that the doses were actually given.

#### Patient evaluation

Clinical examination before and following treatment was performed by a physician who was unaware of treatment allocations. Clinical and parasitological evaluation and monitoring for adverse events were done at the following times: before treatment (day 0; that is, the day treatment began) and at 1, 2, 3, 7, 14, 21, 28, 35 and 42 days after start of treatment. Clinical evaluation consisted of physical examination and measurement of body temperature, heart and respiratory rates. Side effects were defined as symptoms and signs that first occurred or became worse after treatment started and were checked for at every visit. Laboratory tests to further elicit adverse events were not performed routinely at every visit. Any new events occurring during treatment were also considered as side effects.

#### Parasitological evaluation

Thick and thin blood films prepared from finger prick were stained with Giemsa and examined by light microscopy under oil immersion objective lens at 1000 × magnification by two assessors who did not know the drug regimen of the patients. A senior member of the study team reviewed the slides if there was any disagreement between the two microscopists. In addition, the slide of every fourth child enrolled in the study was reviewed by the senior member. Thick and thin blood films, obtained from each child as soon as they came to the clinic onto blood slides, were carefully labelled with the patients’ codes and air-dried before being stained.

Parasitaemia, asexual or sexual, in thick films were estimated by counting asexual and sexual parasites relative to 500 leukocytes, or 500 asexual or sexual forms whichever occurred first. From this figure, the parasite density was calculated assuming a leukocyte count of 6000 μL^−1^ of blood [21–23]. A slide was considered parasite negative if no asexual or sexual parasite was detected after examination of 200 microscope fields.

#### Haematological evaluation

Capillary blood collected before treatment and during follow-up was used to measure haematocrit using a microhaematocrit tube and microcentrifuge (Hawksley, Lancing, UK). Anaemia was defined as a haematocrit <30 % [2, 24]. Early fall in haematocrit to <30 % [or early-appearing anaemia (EAA)] was defined as anaemia occurring within 2 weeks of starting treatment. Late fall in haematocrit to <30 % [or late-appearing anaemia (LAA)] was defined as anaemia occurring after 2 weeks of starting treatment. Initial anaemia recovery time (in anaemic patients at presentation) was defined as time elapsing from start of drug administration to attainment of a haematocrit value ≥30 % [25, 26]. In patients who had EAA or LAA, anaemia recovery time was defined as time from appearance of, to recovery from, anaemia. Fall in haematocrit (FIH) per 1000 parasites cleared from peripheral blood following treatment [FIH/1000 asexual parasites cpb] was defined as relative difference in haematocrit at baseline (pre-treatment) and the first 1 or 2 days after treatment began as numerator, and the corresponding relative difference in parasitaemia as the denominator, and expressing it per 1000 asexual parasites cleared from peripheral blood.

#### Evaluation of temporal changes in haematocrit following treatment

As previously described and in order to determine the different temporal changes in haematocrit following treatment, haematocrit <30 % (anaemia) and ≥30 % (no anaemia) were the reference points in all classified patterns [20]. These patterns, 8 in all, have been described elsewhere [20]. Three of these patterns namely 3, 4 and 7 are associated with late-appearing anaemia [20].

#### Evaluation of response to treatment

Response to drug treatment was assessed using a modified version of the World Health Organization in vivo clinical classification criteria [27]. The clinical classification system consisted of the following categories of response: adequate clinical and parasitological response (ACPR), late parasitological failure (LPF), late clinical failure (LCF), and early treatment failure (ETF). The primary outcomes were the 42-day uncorrected and PCR-corrected efficacy. The secondary outcomes were the fever clearance time, parasite clearance time and recovery from malaria-associated anaemia.

The cure rates on days 28 and 42 were adjusted on the basis of the PCR (polymerase chain reaction) genotyping results of paired samples of patients with recurrent parasitaemia after day 7 of starting treatment as previously described [28]. Fever clearance time (FCT) in patients with presenting body temperature ≥37.5 °C was defined as time elapsing from start of treatment until temperature fell below 37.5 °C and remained so for at least 48 h. Parasite clearance time (PCT) was defined as time elapsing from start of drug administration until there was no patent parasitaemia for at least 72 h. Asexual parasite reduction ratio (PRR) [29] was defined as the ratio of day 0/day 2 parasitaemia (and for convenience, referred to as PRR_D2_). Asexual parasite reduction ratio on day 1 (PRR_D1_) was defined as the ratio of day 0/day 1 parasitaemia.

#### Diagnosis of late-appearing anaemia

A diagnosis of late-appearing anaemia was made if the following criteria were met following initiation of artemisinin-based combination treatments: adequate clinical and parasitological response (ACPR) [27] occurring within 1 week, haematocrit ≥ 30 % at 1 and/or 2 weeks, a fall in haematocrit to <30 % occurring at 3–6 weeks, absence of concomitant illness at 1–6 weeks, and absence of asexual parasitaemia by both microscopy and PCR at 1–6 weeks.

### Statistical analysis

Data were analyzed using version 6 of Epi-Info software [30] and the statistical program SPSS for Windows version 20.0 [31]. Variables considered in the analysis were related to the densities of *P. falciparum* asexual and sexual forms. Proportions were compared by calculating *χ*^2^ using Yates’ correction, Fisher’s exact or Mantel Haenszel tests. Normally distributed, continuous data were compared by Student’s t test and analysis of variance (ANOVA). Data not conforming to a normal distribution were compared by the Mann-Whitney U tests and the Kruskal Wallis tests. The relationship between two variables that are continuous and normally distributed, and those that are discrete and not normally distributed were evaluated by Pearson correlation coefficient and Spearman’s rank correlation coefficient, respectively. A stepwise multiple logistic regression model was used to test the association between late-appearing anaemia or recovery from late-appearing anaemia and factors that were significant at univariate analysis: age, fever 1 day after treatment began, haematocrit <25 % at presentation, haematocrit <30 % 1 day after treatment began, parasite reduction ratio and splenomegaly at enrolment (for late-appearing anaemia), and parasite reduction ratio 1 day after treatment began, parasite clearance time and late-appearing anaemia occurring after 28 days of start of treatment (for recovery from late-appearing anaemia). Because the study was conducted over a period of 7 years, time in years since the commencement of the study was included as a dichotomous covariate in the model for late-appearing anaemia. *P* values of <0.05 were taken to indicate significant differences. Data were double entered serially using patients’ codes and were only analyzed at the end of the study.

## Results

### Characteristics of patients at enrolment

During the study period, a total of 14,564 children with symptoms suggestive of uncomplicated falciparum malaria were screened by microscopy for *Plasmodium falciparum*. Parasitaemia was present in 5926 children. One thousand six hundred and seventy three children met the inclusion criteria but only 624 children were enrolled. Of the 624 children enrolled, 15 children were excluded for the following reasons: self-medication, withdrawal of consent or relocation from study area. All 609 children were seen at all 9 follow-up visits (*n* = 240) or at 8 of 9 follow-up visits (*n* = 369). The characteristics of 609 children enrolled are shown in Table 2. Mean duration of illness before presentation was similar in those <5 and ≥5 years old [2.7 days (95 % CI 2.4–2.8, range 1–7) versus 2.9 days (95 % CI 2.7–3.0, range 1–7), *P* = 0.65].

**Table 2:** Demographic characteristics of enrolled children at presentation

Parameter	Value
No. of children	609
Male (%)	339 (56)
Age < 5 years (%)	165 (27)
5–10 years (%)	331 (54)
> 10 years (%)	113 (19)
Temperature > 37.4 °C (%)	445 (73)
Parasitaemia	
> 100,000 μL^−1^ (%)	179 (29)
> 250,000 μL^−1^ (%)	50 (8)
Haematocrit	
< 30 % (%)	151 (25)
< 15 % (%)	0 (0)
Enlarged liver (%)	98 (16)
Enlarged spleen (%)	64 (11)
Mean value (range) for	
Age (year)	7.1 (0.5–15)
Weight (kg)	19.5 (6–59)
Duration of illness (day)	2.8 (1–7)
Temperature (°C)	38.2 (35.1–41.1)
Haematocrit (%)	32.1 (17–47)
GMPD (μL^−1^ of blood)	55,705 (1636–1,096,636)
Liver enlargement (cm) [*n* = 98]	3.0 (0.1–6.3)
Spleen enlargement (cm) [*n* = 64]	2.6 (0.3–9)

#### Recovery from malaria-associated anaemia at presentation and shortly after presentation

Of the 609 children, 151 children (25 %) were anaemic (haematocrit < 30 %) at presentation. Anaemia was mild (haematocrit 21–29 %) or moderate (haematocrit 15–20 %) in 144 or 7 children, respectively. No child had severe anaemia (haematocrit < 15 %) at presentation. Of the 151 children who were anaemic at presentation, all but 5 recovered from their malaria-associated anaemia. Mean recovery time was 10.6 days (95 % CI 9.5–11.8). Anaemia was mild on day 42 in all children who did not recover from their anaemia.

Of 458 children who were not anaemic at presentation, anaemia developed within the first 3 days of treatment in 147 children and it was mild or moderate in 144 or 3 children, respectively. All children recovered from their anaemia [mean recovery time 8.1 days (95 % CI 7–9.3)].

### Clinical features of children with late-appearing anaemia

#### Frequency and age distribution of children with late-appearing anaemia

**Fig. 1:**
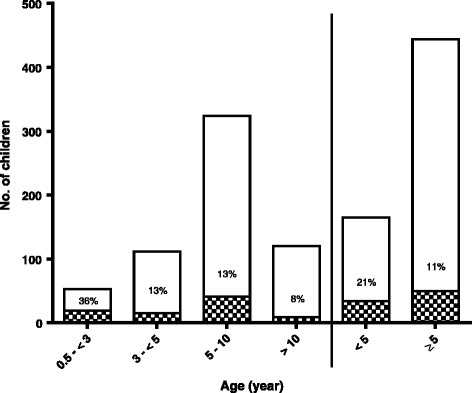
Frequencies and age distribution of children with uncomplicated falciparum malaria who developed late-appearing anaemia following artemisinin-based combination treatments

All 609 children were evaluated for late-appearing anaemia by clinical and parasitological evaluations. Of these, 84 children (14 %) had late-appearing anaemia. Late-appearing anaemia occurred in 54 of 389 (14 %), 25 of 161 (16 %) and 5 of 59 (8 %) children treated with artesunate-amodiaquine, artemether-lumefantrine and dihydroartemisinin-piperaquine, respectively (*P* = 0.41). The frequency of late-appearing anaemia decreased significantly with increasing age (36 %, 13 %, 13 % and 8 % in < 3 years, 3–4 years, 5–10 years and > 10 years, respectively (*P* < 0.0001) (Fig. 1). Late-appearing anaemia was more frequent in children < 5 years old than in those ≥ 5 years old [34 of 165 (21 %) versus 50 of 444 (11 %), *P* = 0.005] (Fig. 1).

#### Temporal changes in haematocrit in children who subsequently developed late-appearing anaemia and comparison of symptoms and signs at presentation in children who subsequently did or did not develop late-appearing anaemia

**Fig. 2:**
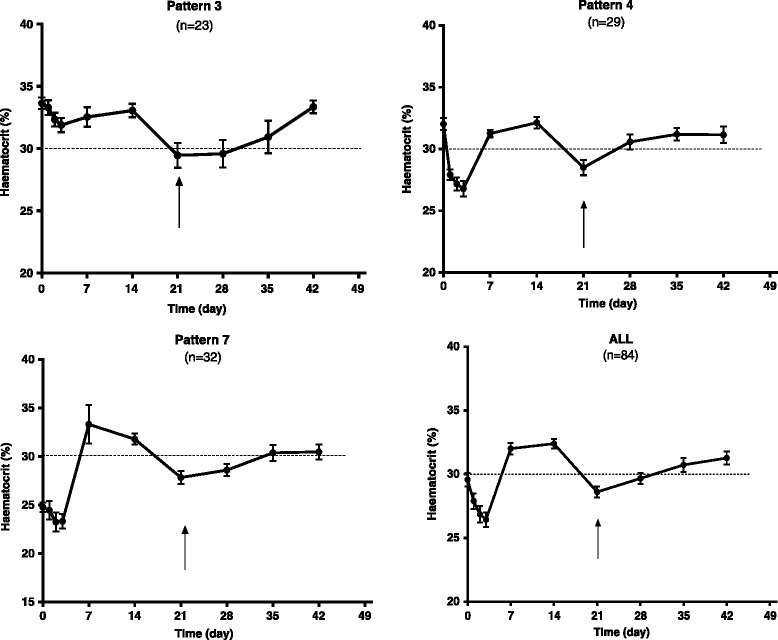
Temporal patterns of change in haematocrit in 84 malarious children who subsequently developed late-appearing after artemisinin-based combination treatments of uncomplicated infections. Arrows indicate time of late-appearing anaemia

Of the 458 children with normal haematocrit at presentation, 216 children had anaemia occurring within 2 weeks of starting treatment. Two hundred and fourteen children recovered from their early-appearing anaemia (mean anaemia recovery time was 8.5 days (95 % CI 7.6–9.4)]. Two children did not recover from their early-appearing anaemia [early-appearing anaemia without recovery]. Of the 214 children who recovered from their early-appearing anaemia, 23 children proceeded to late-appearing anaemia. Figure 2 shows that all 84 children who subsequently developed late-appearing anaemia 3–6 weeks after commencement of treatments can be classed into 3 of the 8 patterns of temporal changes in haematocrit recently described following artemisinin-based combination treatments [20] namely: (i) non-anaemic children at presentation who developed anaemia 3–6 weeks after start of treatment [Pattern 3, *n* = 23]; (ii) non-anaemic children at presentation who developed and recovered from their anaemia within 1 and/or 2 weeks and who subsequently developed anaemia 3–6 week after start of treatment [Pattern 4, *n* = 29]; and (iii) anaemic children at presentation who recovered from their anaemia within 1 and/or 2 weeks and who subsequently developed anaemia 3–6 weeks after start of treatment [Pattern 7, *n* = 32]. The overall pattern (see, ALL in Fig. 2) is consistent with Pattern 7.

**Fig. 3:**
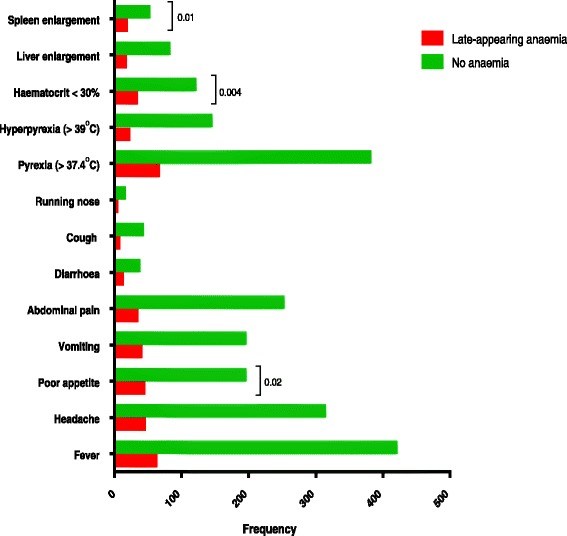
Signs and symptoms at presentation in malarious children who subsequently did or did not develop late-appearing anaemia after artemisinin-based combination treatments

Data regarding signs and symptoms at the time of presentation were available in all 609 children. Poor appetite (43 of 84 (51 %) versus 194 of 525 (39 %), *P* = 0.02), a haematocrit <30 % (32 of 84 (38 %) versus 119 of 525 (23 %), *P* = 0.004) and an enlarged spleen (17 of 84 (20 %) versus 51 of 525 (10 %), *P* = 0.01) were significantly more frequently found at presentation in children who subsequently developed late-appearing anaemia compared to those who did not develop late-appearing anaemia (Fig. 3).

Table 3 shows that children with anaemia at presentation followed by recovery from malaria-related anaemia and who subsequently developed late-appearing anaemia 3–6 weeks after start of treatment were significantly younger, and had significantly higher proportion of children with parasitaemia >100,000 μL^−1^ compared with children who did not have anaemia at presentation. They also had significantly higher parasitaemia at presentation and significantly lower FIH/1000 asexual parasites cleared from peripheral blood compared to children who were not anaemia at presentation but subsequently developed anaemia within a few days of presentation.

**Table 3:** Clinical, parasitological and other characteristics, and temporal patterns of change in haematocrit in children who developed late-appearing anaemia 3–6 weeks after start of treatment

	^a^Pattern 3 (*n* = 23)	^b^Pattern 4 (*n* = 35)	^c^Pattern 7 (*n* = 32)	ALL (*n* = 84)	*P* value
% of total	27	35	38	100	
Gender M/F	14/9	15/14	18/14	47/37	0.80
Age (year)					
Mean (sd)	7.5(3.2)	6.1(3.3)	4.5(2.9)	5.9(3.4)	0.003
Range	2–13	1–13	1.1–13	1–13	0.01
No. <5 years (%)	5 (22)	10 (34)	19 (59)	34 (0.40)	
Duration of illness (days)					
Mean (sd)	2.4(1.1)	3.0(1.0)	2.9(1.2)	2.8(1.1)	0.22
Range	1–5	1–5	1–7	1–7	
Weight (kg)					
Mean (sd)	19.2(5.9)	17.4(5.7)	13.5(4.8)	16.5(5.9)	0.001
Range	9–35	7–29	6–27	6–35	
Haematocrit (%)					
Mean (sd)	33.6(2.2)	32(2.6)	24.8(3.1)	29.6(4.8)	<0.0001
Range	30–37	30–38	18–29	18–38	
Temperature (°C)					
Mean (sd)	37.9(1.2)	38.5(1.0)	38.3(1.3)	38.3(1.2)	0.22
Range	35.3–40	36.2–40.6	35.9–40.5	35.3–40.6	0.11
No. with temp. >37.4 °C (%)	16 (70)	26 (90)	22 (69)	64 (76)	
Parasitaemia (μL^−1^)					
Geometric Mean	61,756	35,466	77,324	53,464	0.049^d^
Range	2880–281,538	1800–345,600	3625–467,681	1800–467,681	0.03
No. with >100,000 μL^−1^(%)	4(17)	6(21)	15(47)	25(30)	
FIH/1000 asexual parasite cpb					
Median	0.015	0.06	0.011	0.023	0.004^e^
Range	0.004–0.35	0.01–0.66	0.0009–0.36	0.0009–0.66	

*sd* standard deviation, *FIH* fall in haematocrit, *cpb* cleared from peripheral blood

^a^Haematocrit ≥30 % at presentation followed by a fall to <30 % 3–6 after start of treatment [Pattern 3]

^b^Haematocrit ≥30 % at presentation, followed by a fall to <30 % within a few days and recovery within 1 and/or 2 weeks, and subsequent fall to <30 % 3–6 weeks after start of treatment. [Pattern 4]

^c^Haematocrit <30 % at presentation, followed recovery within 1 and/or 2 weeks, and subsequent fall to <30 % 3–6 weeks after start of treatment [Pattern 7]

^d^Comparison showed that geometric parasitaemia was similar in Patterns 3 and 7

^e^Comparison showed that median FIH/1000 asexual parasites cleared from peripheral blood was similar in Patterns 3 and 7

#### Time elapsing from presentation to development of late-appearing anaemia

Overall, mean time elapsing from commencement of treatment to late-appearing anaemia was 27.1 days (95 % CI 25.3–28.9, range 21–42), and it was similar in children treated with artesunate-amodiaquine, artemether-lumefantrine or dihydroartemisinin-piperaquine [26.6 days (95 % CI 24.1–28.4, range 21–42), 28.3 days (95 % CI 24.6–31.4, range 21–42) or 31.5 days (95 % CI 17.6–45.9, range 21–42), *P* = 0.38].

Mean of time elapsing from commencement of treatment to late-appearing anaemia was similar in patients with or without anaemia at presentation [27.5 days (95 % CI 24.5–30.5, range 21–42, *n* = 34) versus 26.8 days (95 % CI 24.6–29, range 21–42, *n* = 50), *P* = 0.71]. In children who were anaemic at presentation, mean of time elapsing between recovery from the anaemia at presentation and the onset of late-appearing anaemia was 20.5 days (95 % CI 16.3–24.7, range 14–41).

#### Comparison of symptoms and signs at presentation and during late-appearing anaemia in children with late-appearing anaemia

Of the 84 children with late-appearing anaemia, 78 (93 %) reported no symptoms during late-appearing anaemia. The frequencies of symptoms on presentation and during late-appearing anaemia after artemisinin-based combination treatments are summarised in Table 4. Compared with the symptoms at presentation, late-appearing anaemia was accompanied by significantly fewer symptoms and sign of fever, and absence of liver or spleen enlargement.

**Table 4:** Frequencies of symptoms and signs at presentation and during late-appearing anaemia among 84 malarious children

Symptoms and signs	No of children with symptoms or signs		*P* value
	At presentation (*n* = 84)	During late-appearing anaemia (*n* = 84)	
Fever	61 [73]^a^	3 [4]	<0.0001
Headache	44 [52]	0 [0]	-
Poor appetite	43 [51]	0 [0]	-
Vomiting	39 [46]	1 [1]	<0.0001
Abdominal pain	33 [39]	2 [2]	<0.0001
Diarrhoea	11 [13]	3 [4]	0.047
Cough	6 [7]	3 [4]	0.5
Running nose	3 [4]	0 [0]	-
Weakness	2 [2]	0 [0]	-
Body temperature			
> 37.4 °C	65 [77]	7 [8]	<0.0001
> 39 °C	21 [25]	2 [2]	<0.0001
Liver enlargement	14 [17]	0 [0]	-
Spleen enlargement	17 [20]	0 [0]	-

^a^, [%]

#### The late-appearing anaemia and recovery from late-appearing anaemia

The late-appearing anaemia was mild (haematocrit 21–29 %) in 77 children (92 %), moderate (haematocrit 15–20 %) in 6 children (7 %) or severe (haematocrit <15 %) in 1 child (1 %). Briefly, the child with severe form of late-appearing anaemia was a male, aged 6 years old, with duration of illness of 2 days before presentation, hyperparasitaemia (454,875 μL^−1^) and a haematocrit of 26 % at presentation. Parasitaemia and fever cleared within 1 and 2 days, respectively, after start of treatment with artemether-lumefantrine. Anaemia recovery time from malaria-related anaemia at presentation was 7 days after start of treatment. FIH/1000 asexual parasites cleared from peripheral blood was 0.004. The child developed late-appearing anaemia 3 weeks after start of treatment and was asymptomatic at the time of and during late-appearing anaemia. Recovery from late-appearing anaemia was 14 days and was uneventful.

Fifty six of 84 children recovered from their late-appearing anaemia. Overall, mean recovery time from late-appearing anaemia was 11.8 days (95 % CI 10.8–13.3; range 7–28) and it was similar in children with or without anaemia at presentation [13.5 days (95 % CI 10.8–16.2, range 7–28; *n* = 19) versus 11.2 days (95 % CI 9.3–13.1, range 7–21; *n* = 37), *P* = 0.19]. In the 28 children who did not recover, anaemia was mild in 26 children and moderate in 2 children. The only child with severe anaemia recovered within 14 days (see above).

#### Nadir haematocrit during late-appearing anaemia

Nadir haematocrit was reached on days 21, 28, 35 or 42 in 39 (46 %), 17 (20 %), 14 (17 %) or 14 (17 %) children, respectively. Overall, mean nadir haematocrit was 26.3 % (95 % CI 25.6–26.9; range 14–29) and was similar in those who were anaemic and those who were not anaemic at presentation [25.6 % (95 % CI 24.4–26.8, range 14–29) versus 26.8 % (95 % CI 26–27.5, range 20–29), *P* = 0.08]. There was no correlation between fall in haematocrit/1000 asexual parasites cleared from peripheral blood and nadir haematocrit in the same patients (*r* = 0.11, *P* = 0.37).

Overall, percentage fall, from baseline, in nadir haematocrit was 11.2 % (95 % CI 10.3–12.1, *n* = 273). Percentage fall, from baseline, in nadir haematocrit was significantly higher in children with late-appearing anaemia compared with those without late-appearing anaemia who had falls in their haematocrit 3–6 weeks following treatment [17 % (95 % CI 14.0–19.9, *n* = 56) versus 9.7 % (95 % CI 8.9–10.5, *n* = 217), *P* < 0.0001].

In the 32 children who were anaemic at presentation and who subsequently developed late-appearing anaemia, nadir haematocrit was similar to pre-treatment haematocrit [25.6 % (95 % CI 24.4–26.8) versus 24.8 % (95 % CI 23.7–25.9), *P* = 0.56]. Using day 14 haematocrit as ‘baseline’ haematocrit in children who were anaemic at presentation and who subsequently developed late-appearing anaemia, nadir haematocrit was significantly lower than day 14 haematocrit [25.6 % (95 % CI 24.4–26.8) versus 31.8 % (95 % CI 29.4–34.2), *P* < 0.0001]. Percentage fall in nadir haematocrit using day 14 haematocrit as ‘baseline’ was 16.7 % (95 % CI 9.2–24.3).

### Factors contributing to late-appearing anaemia

#### Factors associated with late-appearing anaemia at presentation

Late-appearing anaemia was significantly more frequent in children who were anaemic at presentation compared to those who were not anaemic at presentation [32 of 151 (21 %) versus 52 of 458 (11 %); *P* = 0.003]. Factors at presentation associated with late-appearing anaemia after artemisinin-based combination treatments are presented in Table 5. An age less than 3 years, fever 1 day after treatment began, haematocrit <25 % at presentation, haematocrit <30 % 1 day after treatment began, parasite reduction ratio >10^4^ 2 days after treatment began, and splenomegaly at enrolment were related to late-appearing anaemia. Gender, duration of illness before presentation, body temperature at presentation, parasitaemia >50,000 μL^−1^ at presentation, parasite reduction ratio 1 day after treatment began, liver enlargement, FIH/1000 asexual parasites cleared from peripheral blood, other measures of therapeutic responses, and year of enrolment were not related to late-appearing anaemia.

**Table 5:** Risk factors for late-appearing anaemia after 2 weeks of starting treatment of uncomplicated falciparum malaria with artemether-lumefantrine, artesunate-amodiaquine or dihydroartemisinin-piperaquine showing the crude and adjusted odds ratio (OR) and the 95 % confidence interval (CI)

Variable	Total no.	No. with late-appearing anaemia	OR (95 % CI)	*P* value	AOR (95 % CI)	*P* value
Gender						
Female	270	37	1			
Male	339	47	1.0(0.6–1.6)	0.95	-	-
Age (years)						
≥ 3	556	65	1		1	
< 3	53	19	4.2(2.3–7.8)	0.000004	2.6 (1.3–5.2)	0.005
Duration of illness (days)						
≤ 2 days	200	28	1			
> 2 days	343	53	1.1(0.7–1.8)	0.81	-	-
Enrolment body temperature						
≤ 37.4 °C	164	19	1			
> 37.4 °C	445	65	1.3 (0.8–2.3)	0.41	-	-
Fever on day 1						
Absent	568	70	1		1	
Present	41	14	3.7(1.8–7.4)	0.0002	3.8 (1.8–8.2)	<0.0001
Haematocrit at presentation (day 0)						
≥ 25 %	554	65	1		1	
< 25 %	55	19	4.0(2.2–7.3)	0.000008	2.2(1.3–3.7)	0.003
Haematocrit on day1						
≥ 30 %	421	40	1		1	
< 30 %	188	44	2.9(1.8–4.7)	0.000009	2.1(1.0–4.3)	0.04
Parasitaemia (μL^−1^)						
≤ 50,000	320	34	1			
> 50,000	289	50	0.7(0.5–1.2)	0.21		
Parasite reduction ratio on day1						
≤ 10,000	443	57	1			
> 10,000	166	27	0.8 (0.5–1.3)	0.34	-	-
Parasite reduction ratio on day2						
≤ 10,000	532	67	1		1	
> 10,000	77	17	2.0 (1.1–3.6)	0.038	2.1(1.1–3.9)	0.03
Liver enlargement						
Absent	511	70	1			
Present	98	14	1.0(0.6–1.9)	0.98		
Spleen enlargement						
Absent	535	67	1		1	
Present	64	17	2.4(1.3–4.3)	0.008	2.0(1.1–3.9)	<0.0001
Parasite clearance time						
< 1 day	506	73	1			
> 1 day	103	11	0.7(0.4–1.4)	0.40	-	-
Fever clearance time						
< 1 day	574	75	1			
> 1 day	35	9	1.9(0.9–4.3)	0.13		
FIH/1000 asexual parasite cpb^a^						
≥ 0.05	133	28	1			
< 0.05	258	39	0.7(0.4–1.2)	0.27		
Year of enrolment						
< 2010	242	33	1			
≥ 2010	367	51	1.0(0.6–1.6)	0.93		

^a^cpb, cleared from peripheral blood

#### Risk factors for late-appearing anaemia

In the multivariate analysis, an age less than 3 years, fever 1 day after treatment began, haematocrit <25 % at presentation, haematocrit <30 % 1 day after treatment began, parasite reduction ratio >10^4^ 2 days after treatment began and splenomegaly at enrolment were independent risk factors for late-appearing anaemia (Table 5).

#### Risk factors for unresolved late-appearing anaemia

Of the 84 children with late-appearing anaemia, 28 (33 %) did not recover from their late-appearing anaemia during the 42-day follow-up period. The proportions of the children who did not recover were similar in the 3 treatment groups: 18 of 55 (33 %) in artesunate-amodiaquine, 7 of 25 (28 %) in artemether-lumefantrine and 3 of 4 (75 %) in dihydroartemisinin-piperaquine treatment groups [*P* =0.18]. Factors associated with unresolved late-appearing anaemia were parasite reduction ratio ≤10^4^ 1 day after treatment began and occurrence of late-appearing anaemia after 28 days of starting treatment (Table 6). In a multivariate analysis, occurrence of late-appearing anaemia after 28 days of starting treatment was the only independent predictor of unresolved late-appearing anaemia (adjusted odd ratio [AOR] = 7.5, 95 % CI 2.5–22.9, *P* <0.0001; Table 6) during the 42-day follow-up period.

**Table 6:** Risk factors for unresolved late-appearing anaemia after 2 weeks of starting treatment of uncomplicated falciparum malaria with artemether-lumefantrine, artesunate-amodiaquine or dihydroartemisinin-piperaquine showing the crude and adjusted odds ratio (OR) and the 95 % confidence interval (CI)

Variable	Total no.	No. with late-appearing anaemia	OR (95 % CI)	*P* value	AOR (95 % CI)	*P* value
Gender						
Male	47	16	1			
Female	37	12	0.9(0.4–2.3)	1.00	-	-
Age (years)						
≥ 3	65	20	1			
< 3	19	8	1.6(0.6–4.7)	0.52	-	-
Haematocrit at presentation (day 0)						
≥ 25 %	65	20	1			
< 25 %	19	8	1.6(0.6–4.7)	0.52	-	-
Parasitaemia (μL^−1^)						
≤ 50,000	34	13	1			
> 50,000	50	15	0.7(0.3–1.7)	0.58		
Parasite reduction ratio on day 1						
> 10^4^	57	14	1		1	
≤ 10^4^	27	14	3.3 (1.3–8.7)	0.026	2.9 (1. −8.5)	0.05
Fever clearance time						
≤ 1 day	75	25	1			
> 1 day	9	3	1.0(0.2–4.3)	1.00		
Time of occurrence of late-appearing anaemia						
≤ 28 days	62	13	1		1	
> 28 days	22	15	8.1(2.7–22.9)	0.0002	7.5(2.5–22.9)	<0.0001

### Therapeutic responses according to drug treatment

In the initial comparison of artesunate-amodiaquine and artemether-lumefantrine (*n* = 492), parasite prevalence 1 day after treatment began and parasite clearance time were significantly lower in children treated with AA compared to AL (Table 7). In the later study (*n* = 117), therapeutic responses were similar in children treated with AA, AL or DHP (Table 7).

**Table 7:** Baseline characteristics and outcomes of treatments in malarious children treated with artesunate-amodiaquine, artemether-lumefantrine or dihydroartemisinin-piperaquine

Parameters	Initial study^a^			Later study^b^			
	AA (*n* = 360)	AL (*n* = 132)	*P* value	AA (*n* = 29)	AL (*n* = 29)	DHP (*n* = 59)	*P* value
Age (year)							
Mean (95 % CI)	7.1(6.8–7.5)	6.1 (5.5–6.6)	0.001	6.8 (5.3–8.3)	7.5 (6.1–8.9)	9.5 (8.4–10.5)	0.01
No <5 years (%)	85 (24)	50 (38)	0.002	11 (38)	10 (34)	10 (17)	0.06
Body temperature (°C)							
Mean (95 % CI)	38.3 (38.2–38.4)	38.4 (38.2–38.6)	0.37	38 (37.5–38.4)	37.8 (37.2–38.3)	37.7 (37.2–38.3)	0.76
No. ≥ 37.4 °C (%)	283 (79)	100 (76)	0.58	15 (52)	16 (55)	32 (54)	0.96
Haematocrit (%)							
Mean (95 % CI)	32.3 (31.9–32.8)	31.2 (30.5–32)	0.02	30.3 (29.2–32.3)	31.5 (29.7–33.2)	33.4 (33.2–34.5)	0.01
No. < 30 % (%)	78 (22)	45 (34)	0.01	11 (38)	6 (21)	11 (19)	0.12
Parasitaemia (μL^−1^)							
Geometric mean	59,978	60,210	0.48	55,803	45,054	33,543	0.07
Range	1636–1,096,636	1657–900,532		2100–249,600	2220–454,875	2000–458,750	
No. > 100,000 μL^−1^ (%)	107(30)	43 (33)	0.62	10 (34)	8 (28)	13 (22)	0.46
No. > 100,000 μL^−1^ (%)	30 (8)	13 (10)	0.73	0 (0)	3 (10)	4 (7)	0.68
No. with parasitaemia on day 1 (%)	22 (6)	23 (17)	0.05	14 (48)	15 (52)	28 (47)	0.93
Parasite positivity on day 3 (%)	2 (0.6)	1 (0.7)	1.0	0 (0)	0 (0)	0 (0)	-
PRR_D1_							
Geometric mean	3.3 × 10^4^	1.6 × 10^4^	0.27	2.4 × 10^3^	1.3 × 10^3^	1.9 × 10^4^	0.84
Range	2.0 × 10^0^-1.1 × 10^6^	2.1 × 10^0^-9.0 × 10^5^		3.3 × 10^0^-2.4 × 10^6^	7.1 × 10^−1^-4.5 × 10^5^	5.6 × 10^−1^-1.3 × 10^5^	
PRR_D2_							0.08
Geometric mean	5.7 × 10^4^	6.0 × 10^4^	0.4	4.1 × 10^4^	4.5 × 10^4^	2.6 × 10^4^	
Range	1.6 × 10^1^-1.1 × 10^6^	1.6 × 10^3^-9.0 × 10^5^		2.1 × 10^3^-2.4 × 10^6^	2.2 × 10^3^-4.5 × 10^5^	3.5 × 10^1^-4.6 × 10^5^	
Parasitological efficacy							
ACPR	348	136		27	28	59	
LPF	12	4		2	1	0	
LCF	0	0		0	0	0	
ETF	0	0		0	0	0	
Cure rate (%)	98.7	97.1	1.0	93.1	95.8	100	1.0
Fever clearance time (day)							
Mean (95 % CI)	1.1 (1–1.1)	1.1 (1–1.2)	0.28	1.1 (0.9–1.3)	1.3 (1–1.5)	1.1 (1–1.2)	0.3
Parasite clearance time (day)							
Mean (95 % CI)	1.1 (1.05–1.1)	1.2 (1.1–1.3)	0.004	1.6 (1.4–1.9)	1.6 (1.4–1.8)	1.5 (1.4–1.7)	0.84
Anaemia recovery time (day)							
Mean (95 % CI)	11.1 (9.5–12.7)	9.4 (7.3–11.5)	0.22	18.9 (13.1–24.7)	22.8 (21–45)	11.2 (5–17.4)	0.09

*AA* artesunate-amodiaquine, *AL* artemether-lumefantrine, *DHP* dihydroartemisinin-piperaquine, *PRR*_*D1*_ parasite reduction ratio 1 day after treatment started, *PRR*_*D1*_ parasite reduction ratio 2 days after treatment started, *ACPR* adequate clinical and parasitological responses, *LPF* late parasitological failure, *LCF* late clinical failure, *ETF* early treatment failure; ^a^, study conducted between 2008 and 2013, ^b^, study conducted in 2014

### Comparison of therapeutic responses of children with or without late-appearing anaemia

Overall, fever clearance time was 1.1 day (95 % CI 1.0–1.1) and it was significantly faster in children without late-appearing anaemia compared with children who had late-appearing anaemia [1.1 day (95 % CI 1.0–1.1) versus 1.3 day (95 % CI 1.1–1.5), *P* < 0.0001]. Similarly, the proportion of children who remained febrile 1 day after treatment began was significantly lower in children without late-appearing anaemia compared to those with late-appearing anaemia [27 of 525 (5 %) versus 14 of 84 (17 %), *P* < 0.0001]. Parasite clearance time [1.2 day (95 % CI 1.1–1.2) versus 1.2 day (95 % CI 1.1–1.3), *P* = 0.53], geometric mean parasite reduction ratio 1 day after treatment began [1.6 × 10^4^, range 0.6 × 10^0^–1.1 × 10^6^ versus 2.1 × 10^4^, range 2.7 × 10^0^–4.7 × 10^5^, *P* = 0.72) and geometric mean parasite reduction ratio 2 days after treatment began [5.3 × 10^4^, range 1.6 × 10^1^–1.1 × 10^6^ versus 5.0 × 10^4^, range 5.9 × 10^1^–4.7 × 10^5^, *P* = 0.72] were similar in children without late-appearing anaemia and those with late-appearing anaemia. Parasite positivity on day 1 was similar in children without and those with late-appearing anaemia [93 of 525 and 11 of 84, *P* = 0.32].

### Reported adverse events

Twenty of 84 (24 %) children and 147 of 525 (28 %) children with and without late-appearing anaemia, respectively, reported at least 1 adverse event in the first week of starting treatment. There was no significant difference in the proportions reporting adverse events in the 2 groups (*P* = 0.95). In children with late-appearing anaemia, 7 (8 %), 11 (13 %), 3 (4 %), 3 (4 %), 4 (5 %), 7 (8 %), 1 (1 %) and 0 (0 %) children reported abdominal pain, fever, vomiting, weakness, headache, cough, anorexia and dizziness, respectively. In children without late-appearing anaemia, 40 (8 %), 57 (11 %), 21 (4 %), 10 (2 %), 14 (2.7 %), 33 (6 %), 18 (3.3 %) and 6 (1 %) children reported abdominal pain, fever, vomiting, weakness, headache, cough, anorexia and dizziness, respectively. There was no significant difference in the proportions reporting each of these adverse events in the children with late-appearing anaemia and those without late-appearing anaemia.

## Discussion

In this study conducted over a period of seven years, we described the clinical and parasitological features, the patterns of temporal changes in haematocrit and the risk factors for late-appearing anaemia in acutely malarious children resident in an endemic area, following artemisinin-based combination treatments. Using a set of clinical and parasitological criteria showed that a relatively asymptomatic late-appearing anaemia that was predominantly mild in intensity occurred in 14 % of the children with uncomplicated falciparum malaria following artemisinin-based combination treatments. In these children, five factors were independent predictors of late-appearing anaemia at presentation namely an age <3 years, anaemia at presentation and 1 day after treatment began, fever 1 day after treatment began and splenomegaly.

The finding that the late-appearing was not severe in nature supports a recent study from Mali which showed that there was no association between artemisinin-based combination treatments and severe delayed anaemia in children [32]. In that study, treatment with artesunate alone increased the risk relative to other non-artemisinin-based combination treatments [32].

In uncomplicated falciparum infections in children, a variety of temporal patterns of change in haematocrit/haemoglobin can occur following artemisinin-based combination treatments [20, 33]. Failure of haematological recovery is not uncommon following artemisinin-based combination treatments in anaemic and non-anaemic patients. Obonyo and others [33] reported that 76 % of Kenyan children treated with artemisinin-based combination drugs failed to achieve complete haematological recovery 4 weeks following start of treatment. Although, details of temporal changes in haemoglobin were not described, these authors found 10 % of anaemic children achieved temporary haematological recovery followed by failure of recovery during a 4-week period. In addition, they showed that 23 % of children with normal haemoglobin at presentation became anaemic and failed to recover from their anaemia. These 2 patterns are reminiscent of 2 patterns (Pattern 7 and Pattern 4, respectively) of late-appearing anaemia in the 84 children reported in present study. Three of the recently described patterns [20] showing late fall in haematocrit to <30 % occurring 3–5 weeks after start of treatment, were observed in the 84 children with late-appearing anaemia. These patterns occurred with similar frequency following treatment with artesunate-amodiaquine, artemether-lumefantrine or dihydroartemisinin-piperaquine. The overall pattern of haematocrit change in all 84 children is a classical Pattern 7 (anaemia at presentation, followed by recovery, followed by late-appearing anaemia) and it is a confirmation that anaemia at presentation and occurring a few days after presentation are independent predictors of late-appearing anaemia 3–6 after start of artemisinin-based combination treatments in African children (see below).

An interesting pattern not previously described occurred in 2 children in the present study: early monophasic fall in haematocrit to <30 % and without recovery from the anaemia during the 6 weeks period of follow-up. This pattern is reminiscent of the pattern described by Obonyo and others (see above). More studies are now needed to elicit the frequency of this new pattern following artemisinin-based combination treatments.

Young children presenting with anaemia, splenomegaly, those who were anaemic and/or febrile 1 day after treatment started, and those who had rapid clearance of their parasitaemias were 2–4 times more likely to have late-appearing anaemia compared with children without these characteristics. Splenomegaly and hepatomegaly are not uncommon in malarious children with or without anaemia at presentation [34]. Both organs may also not completely regress in a small proportion of children following treatment [34]. It is however, unclear why splenomegaly at presentation in the cohort of children evaluated was an independent risk factor for late-appearing anaemia after 2 weeks of starting treatment.

Artemisinin-based combination treatments often result in high parasite reduction ratio 2 days after treatment began [35]. It was therefore not surprising that children with parasite reduction ratio >10^4^ 2 days after treatment began were 2 times more likely to have late-appearing anaemia compared with children who had parasite reduction ratio ≤10^4^ 2 days after treatment began. Artemisinin-based combination treatments can also prevent precipitous falls in haematocrit shortly after start of treatment particularly when parasitaemias are high because the once-infected red blood cells are preserved and are destroyed 7–21 days later [5, 7]. In the field, prevention of precipitous falls in haematocrit may be measured by estimating fall in haematocrit/1000 asexual parasites cleared from peripheral blood [36]. FIH values are usually low in patients with high parasitaemias suggesting much haematocrit conservation [36]. It was therefore surprising that values of FIH <0.05 and parasite clearance time ≤1 day were not associated with late-appearing anaemia.

It is also not surprising that late-appearing anaemia occurring 4 weeks after start of treatment was the only predictor of failure to recover from anaemia following artemisinin-based combination treatments. The relatively limited period of follow-up of 6 weeks made it impossible to determine the outcomes of recovery (i. e. eventful or uneventful) in these children. In this context, young children at risk of developing late-appearing anaemia would require follow up for period longer than 6 weeks in endemic areas because of the relatively asymptomatic nature of late-appearing anaemia. One of the long term consequences of the asymptomatic nature of late-appearing anaemia is the development of chronic anaemia and its associated complications. In this context, studies are needed to address the asymptomatic nature of late-appearing anaemia.

Two of the many features of delayed anaemia following use of intravenous artesunate are initial conservation of once-infected red blood cells (preventing precipitous fall in haematocrit during treatment) and destruction of the once-infected red blood cells 7–21 days later causing severe haemolytic anaemia measured as 10 % fall in pre-treatment haemoglobin associated with haptoglobin <0.1 g/L and either an increase in LDH to >390 IL/L or a 10 % rise >7 days after start of treatment [7, 10]. In present study, haematocrit conservation was measured as FIH/1000 asexual parasites cleared from peripheral blood [36] and loss of conserved haematocrit 3–5 weeks later was measured as a fall in haematocrit to <30 % in children who were not anaemic in the 1 and/or 2 weeks following successful treatment of falciparum malaria. In these contexts, the limitations of our study using clinical and parasitological criteria for the diagnosis of late-appearing anaemia are: not quantifying the once-infected red blood cells, non-evaluation of laboratory parameters for the confirmation of haemolytic or non-haemolytic nature of the late-appearing anaemia, and not estimating the contribution of the background causes of anaemia in this endemic area (for example helminth infections, malnutrition, the haemoglobinopathies, glucose-6-phosphate dehydrogenase deficiency) to the various patterns of change in haematocrit to <30 % following artemisinin-based combination treatments. In addition, artemisinin-related drugs have been shown to cause reticulocytopaenia in animal studies by supressing erythroblasts [37]. Estimation of reticulocytes and their contributions to the temporal changes in haematocrit and late-appearing anaemia were not evaluated in this study.

The strengths of our study lie in the ability to use simple parameters that can be readily applicable in resource-poor endemic countries for the diagnosis of late-appearing anaemia following artemisinin-based combination treatments and the ability to identify early and to initiate appropriate measures in children who are at risk of developing late-appearing anaemia following artemisinin-based combination treatments. Studies are now needed in children with uncomplicated infections who developed late-appearing anaemia following artemisinin-based combination treatments to: quantify once-infected red blood cells and the kinetics of the disposition of these red blood cells, determine the haemolytic or non-haemolytic nature of the anaemia when it occurs, clinical and parasitological case definition for late-appearing anaemia, the appropriate measures for preventing late-appearing anaemia in children at risk, and the contribution of background anaemia to the late-appearing anaemia in children resident in endemic areas.

There is need to justify the criteria used for the diagnosis of late-appearing anaemia. Since drug insensitivity, recrudescence or reinfections are risk factors for anaemia following antimalarial treatments [2, 24, 32], the criteria for the diagnosis of late-appearing namely: adequate clinical and parasitological response within 1 week of stating treatment, parasite negativity by microscopy and polymerase chain reaction after initial clearance and normal haematocrit 1 and/or 2 weeks after start of treatment limit these risk factors as confounding variables for the diagnosis of late-appearing anaemia. In addition, the findings of the present study namely: children anaemic at presentation and those developing anaemia within the first few days of treatment are at increased risk of late-appearing anaemia strengthens the criterion of normal haematocrit at 1 and/or 2 weeks for the diagnosis of late-appearing anaemia. This criterion ensures that children with anaemia at presentation and who did not achieve haematological recovery are not wrongly diagnosed as having late-appearing anaemia following artemisinin-based combination treatments.

## Conclusion

In conclusion, a relatively asymptomatic late-appearing anaemia with uneventful recovery can occur in young malarious children following artemisinin-based combinations. Its occurrence may have implications for case and community management of anaemia and for anaemia control efforts in sub-Saharan Africa where artemisinin-based combination treatments have become first-line antimalarials.

## Abbreviations

%, percent; °C, degree celsius; AA, artesunate-amodiaquine; ACTs, artemisinin-based combination treatments; AL, artemether-lumefantrine; AOR, adjusted odds ratio; cbp, cleared from peripheral blood; DHP, dihydroartemisinin-piperaquine; EAA, early-appearing anaemia; FCT, fever clearance time; FIH, fall in haematocrit; g, gram; GMPD, geometric mean parasite density; HCT, haematocrit; kg, kilogram; L, litre; LAA, late-appearing anaemia; LDH, lactate dehydrogenase; M/F, male/female; mg, milligram; OR, odds ratio; PADH, post artesunate delayed haemolysis; PCR, polymerase chain reaction; PCT, parasite clearance time; sd, standard deviation; μL, microliter
